# A Prescription

**Published:** 1886-09

**Authors:** 


### *A PRESCRIPTION.*

The young physician, newly made
With doctor's stuff and surgeon's blade,
Now for the first, set up his trade.

The sexton smiled with a side way look,
At his rusty spade there on the hook.

Fair village maids ne'er ill before,
Vied in disorders by the score,
And Lydia not behind the rest,
Feigned a queer aching in her breast.

The doctor came—perceived the ruse,
And mixed for her a harmless dose.
But Lydia aching still, made sure
Hers should not be a hasty cure;
Yet since all ills must have an end,
She too, at length, began to mend.

One day in quite a roguish vein,
She teased her doctor in this strain:
"Do tell me, Doctor, if you will,
What's rolled up in this little pill?"

"The pill, ah yes, ahem! the pill
Is only meant for oue who's ill."

"Yes, yes, I know; but Doctor, tell,
What is there here to make me well?"

"Ah! that's a secret only we
Should know who sign ourselves M. D."

"But were *you* sick the same as *I*,
Would *you* for cure on *this* rely?
Or would you other potions try?"

"That I *am sick* the same as *you*,
You know, you little witch, 'tis true,
And truth to say, *my* healing lies
Not in these *pills*, but in your *eyes*."

